# Post-surgery and recovery experiences following one- and two-stage revision for prosthetic joint infection—A qualitative study of patients’ experiences

**DOI:** 10.1371/journal.pone.0237047

**Published:** 2020-08-03

**Authors:** Cecily K. Palmer, Rachael Gooberman-Hill, Ashley W. Blom, Michael R. Whitehouse, Andrew J. Moore

**Affiliations:** 1 National Institute for Health Research, Applied Research Collaboration West (NIHR ARC West), University Hospitals Bristol NHS Foundation Trust and University of Bristol, Bristol, United Kingdom; 2 Population Health Sciences, Bristol Medical School, University of Bristol, Bristol, United Kingdom; 3 Musculoskeletal Research Unit, Translational Health Sciences, Bristol Medical School, University of Bristol, Bristol, United Kingdom; 4 National Institute for Health Research, Bristol Biomedical Research Centre, University Hospitals Bristol NHS Foundation Trust and University of Bristol, Bristol, United Kingdom; University Hospital Zurich, SWITZERLAND

## Abstract

Deep prosthetic hip infection is a devastating complication of hip replacement surgery, and treatment often involves multiple revision surgeries with antibiotic chemotherapy to control the infection. The aim of this study was to explore patients’ experiences of early and longer-term recovery after one-stage or two-stage revision with an excised hip, a temporary cement spacer or a custom-made articulating spacer. We interviewed 32 participants taking part in a surgical trial at two time points (2–4 months and 18 months) following one- or two-stage revision surgery. The analytic approach was inductive using the constant comparative method to generate themes from the data. Participants’ early recovery after revision was characterised by a long hospital stay with burdensome antibiotics and limited physiotherapy provision. Participants undergoing two-stage revision with an excised hip or a cement spacer described severe mobility restrictions which affected all aspects of their lives, while those undergoing one-stage revision, or two-stage revision with an articulating spacer were more mobile and independent, with some limitations. Participants with a cement spacer also reported more pain than other treatment groups, while those with an articulating spacer appeared to perceive that their recovery was slow. At 18 months, participants in all groups described both improvements and losses in mobility and functional ability. Participants in all treatment groups expressed considerable emotional resilience during recovery from revision, which may be linked to opportunities to talk with the trial personnel. Participants identified the need for better information and psychological and physical support. Experience of recovery differs after one- and two-stage revision, and further in relation to the use of spacers. Mobility, function, independence and pain are important aspects of recovery which affect all aspects of day-to-day life. Increased information and more opportunities to talk and share experiences may provide psychological support during recovery.

## Introduction

Approximately 98,000 primary hip replacement operations took place in 2018 in England, Wales and Northern Ireland [1]. Prosthetic joint infection (PJI) is an uncommon but serious complication, affecting 0.4 to 1% of patients after total hip replacement [2, 3]. Over 1,000 revisions are performed annually in the UK for PJI following hip replacement [2].

PJI occurs when microorganisms establish on the surface of the prosthetic joint implant [4]. Symptoms vary but commonly include pain, joint swelling, warmth around the joint, fever, drainage, or the presence of a sinus tract linked to the hip replacement [4]. PJI occurring within one year of joint replacement surgery are thought to be caused by microorganisms introduced during the operation. However, PJI can result from haematogenous spread from infection in another part of the body and thus the risk of PJI remains throughout the life of the implant [4].

Treatment for PJI often involves major revision surgery, commonly either a one-stage or two-stage revision surgery, depending on multiple factors [5]. Revision surgery involves the removal of the infected prosthesis, bone, soft tissue and cement [6], followed by an antibiotic regime. Once all infected material is removed, in a one-stage revision, a new prosthesis is implanted during the same operation. In a two-stage revision, the implantation of the new prosthesis is delayed until a second operation [6]. This period of delay can vary in length, typically from 3–6 months, although it can be much longer [7], and is informed by a complex interplay of individual patient clinical findings, test results, eradication of the infection, social and care needs, service provision and availability of facilities. During the period between the two revision stages, patients may be left with an excised hip joint, creating an effectively empty space. Alternatively they may be fitted with a temporary static or hemiarthroplasty type cement spacer that delivers antibiotics locally to the hip, maintains soft tissue length, and may allow weight bearing [8]. A custom-made articulating spacer (CUMARS) is an option that may be used to increase function between revision stages and allow better mobilisation [5]. CUMARS involves fitting an acetabular component and femoral implant with articulation occurring between these, with the implant loosely cemented making extraction and replacement easier during the second operation [8].

Recent work has found the risk of reinfection to be similar for both one- and two-stage revision [9, 10]. However, there has been little attention paid to outcomes relating to patients’ lived experience of recovery after revision and between stages in two-stage revision [9, 11]. Qualitative research enables a robust and detailed exploration of how patients experience surgical interventions, allowing the consequences for patients’ lives to be understood. It also allows patients to articulate areas of importance to them [12]. A single qualitative study exploring experiences of hip PJI and treatment found that two-stage revision had a greater impact on participants’ well-being and was characterised by long periods of immobility and related psychological distress [7]. Similarly, a study of patients undergoing 2-stage revision for PJI of the knee found patients to experience a considerable psychosocial burden [13]. The aim of this qualitative interview study, embedded within a randomised trial was to explore patient participants’ early and longer-term experiences of recovery within a surgical trial for PJI, and compare reported experiences after one-stage, or two-stage revision with an excised hip (also known as an excision arthroplasty), a non-articulating or hemiarthroplasty cement spacer, or CUMARS to identify possible differences.

## Materials and methods

Patients who had consented to take part in the INFORM randomised surgical trial comparing one- and two-stage revision surgery for prosthetic joint infection of the hip [ISRCTN10956306] were asked if they would be willing to be interviewed about their experiences [11]. The study centres involved in the trial comprised 12 NHS secondary care orthopaedic units in England and Wales. Selected sites were high-volume tertiary referral centres for infected joint replacements or large NHS orthopaedic units. Trial participants were sent an information pack and asked to complete a reply slip if they wished to take part in an interview. The researchers (AJM, CM, CP) contacted potential participants to provide further information about the study and arrange a time for interview if they agreed to participate. Before interview, participants had the opportunity to ask questions about the study, before providing their written consent to audio recording, and publication of anonymised interview extracts. Interviews took place by telephone or in patients’ homes.

Interviews were undertaken by AJM, CM and CP, all experienced qualitative researchers. Topic guides were developed with members of a patient and public involvement group [14] S1 and S2 Files. The topic guide was used flexibly to ensure key topics of interest were covered and to allow participants to discuss areas of importance to them. Interview questions explored experiences of trial participation; revision surgery and care; discharge and recovery; and impact of treatment. The final sample size was determined by achievement of saturation during the iterative analysis process, such that no new themes were emerging from analysis [15, 16].

30 participants agreed to take part in an interview at timepoint one; undertaken two to four months after their first revision operation in the trial. Patients interviewed were recruited from 8 of the 12 study centres. These were Bristol, Cardiff, Exeter, Oswestry, Oxford, Sheffield, Wrightington, and Coventry. Those interviewed were asked if they would be willing to take part in an end of study interview, undertaken 18 months after their first revision operation (timepoint two). The second interview was undertaken at 18 months after revision due to this being the primary outcome timepoint of the randomised trial from which the patients were recruited to the qualitative study, and also to ensure that participants undergoing a two-stage revision had undergone the second stage of their revision, and to allow all participants to reflect on their experience of recovery in the longer-term. Willing participants were later contacted and underwent a second consent process. To ensure a balance of participants undergoing one and two-stage revision, three additional participants, who had not been interviewed at the first timepoint, were invited to take part in an interview at timepoint two, of which two were interviewed. At the 18-month interview, participants who had undergone a two-stage revision were asked about their second operation and the period between revision stages.

The final sample comprised 32 participants (see Table 1), 30 of whom were interviewed at timepoint one (29 by telephone, 1 in person); and 17 at timepoint two (15 in person, 2 by telephone) (see Table 2). Interviews were audio-recorded, transcribed, anonymised and imported into NVivo 11 (QSR International, Melbourne, Australia) qualitative data-management software.

**Table 1:** Participant sample characteristics.

Pseudonym	Gender	Age	Revision type	Length of time between revision stages (patient disclosed at time point two)
Arabella	F	71	One stage	N/A
Colin	M	89	One stage	N/A
Jayne	F	72	One stage	-
Thomas	M	79	One stage	N/A
Freda	F	80	One stage	-
Steve	M	57	One stage	-
Alex	M	64	One stage	-
Frank	M	79	One stage	-
Geoff	M	71	One stage	N/A
Christopher	M	57	One stage	-
Albert	M	62	One stage	-
Jack	M	56	Two stage: excised hip	7 months
Arthur	M	69	Two stage: excised hip	-
Lois	F	62	Two stage: excised hip	-
Anna	F	58	Two stage: excised hip	5 months
Fred	M	51	Two stage: cement spacer	7 months
George	M	73	Two stage: cement spacer	-
Cathy	F	71	Two stage: cement spacer	6 months
Bernie	M	79	Two stage: cement spacer	-
Trevor	M	57	Two stage: cement spacer	-
Janet	F	70	Two stage: cement spacer	-
Elspeth	F	69	Two stage: cement spacer	Not disclosed
Stanley	M	73	Two stage: CUMARS	N/A
Daphne	F	72	Two stage: CUMARS	-
Winnie	F	77	Two stage: CUMARS	N/A
Darren	M	66	Two stage: CUMARS	-
Teresa	F	70	Two stage: CUMARS	-
Sandra	F	74	Two stage: CUMARS	6 months
Ruth	F	77	Two stage: CUMARS	N/A
Enid	F	75	Two stage: CUMARS	4 months
Jenny	F	68	Two stage: CUMARS	N/A
Ian	M	56	Two stage: CUMARS	-

**Table 2:** Interviews completed.

	TOTAL	One-stage	Two-stage: cement spacer between stages	Two-stage: excised hip between stages	Two-stage: CUMARS between stages
First timepoint interview	30	9	7	4	10
Second timepoint interview	17	6	3	2	6

Data collection and analysis took place concurrently. Data were analysed inductively using the constant comparative method, a robust qualitative analysis approach that generates themes from the data [15]. Interview data were descriptively coded, codes were compared and extended or refined. Coded data were then grouped together to develop emerging themes linked to points along the treatment and recovery pathway. Coding of transcripts was shared between two experienced qualitative researchers (AM, CP), with emerging themes discussed and refined. Data from participants undergoing one-stage revision, and those undergoing a two-stage revision who had an excised hip; a temporary cement spacer; or a custom-made articulating spacer (CUMARS), were analysed separately to compare experiences and identify possible differences. Ethics approval was granted by NRES Committee South West, Exeter (14/SW/0072) on 29 April 2014.

## Results

Analysis found commonality and differences in the recovery experiences of participants undergoing one and two-stage revision. During their early recovery period, participants described prolonged stays in hospital, with burdensome antibiotics and brief physiotherapy involvement.

**Fig 1:**
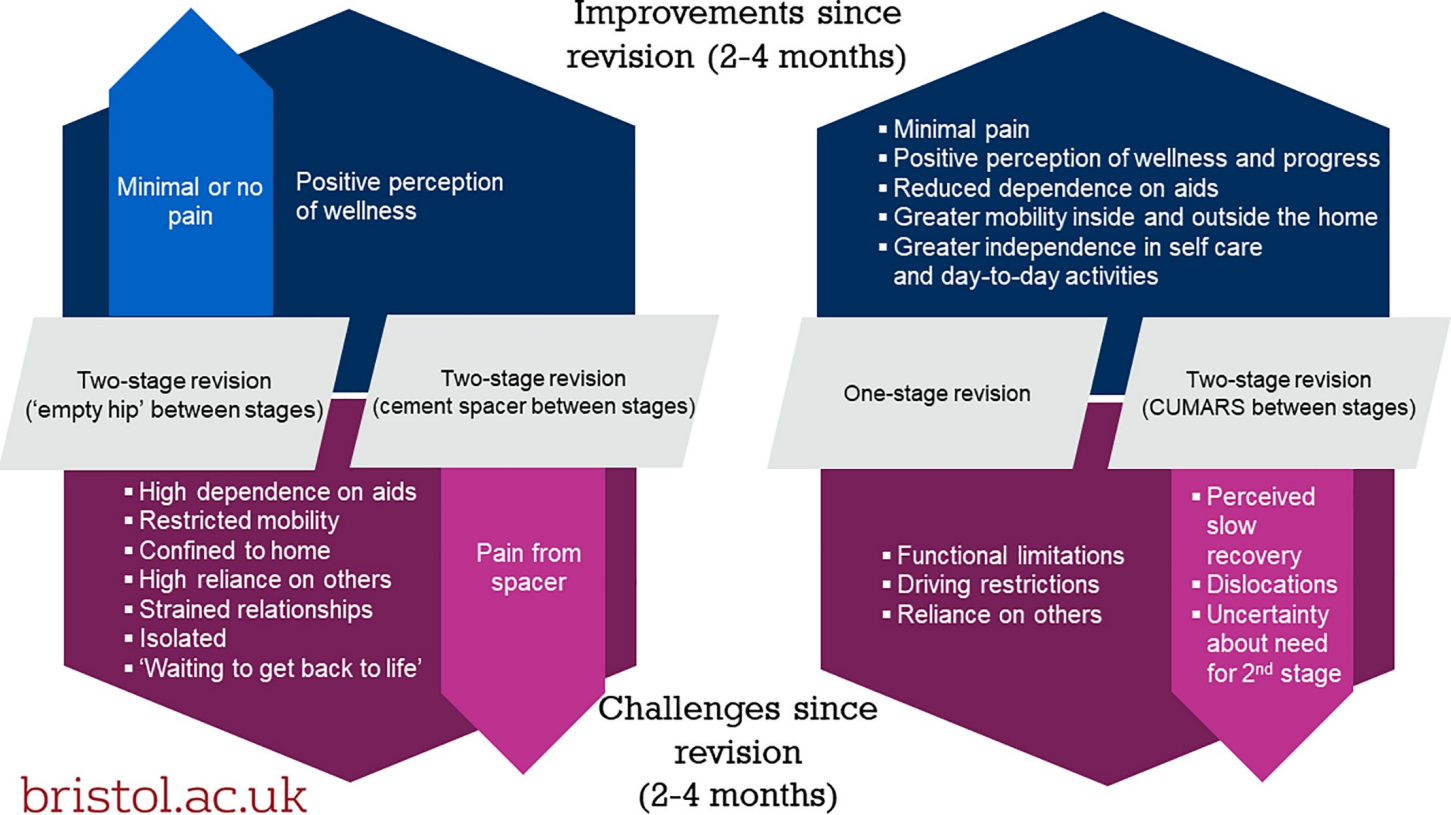
Thematic map of improvements / challenges in patient recovery at 2–4 months.

**Fig 2:**
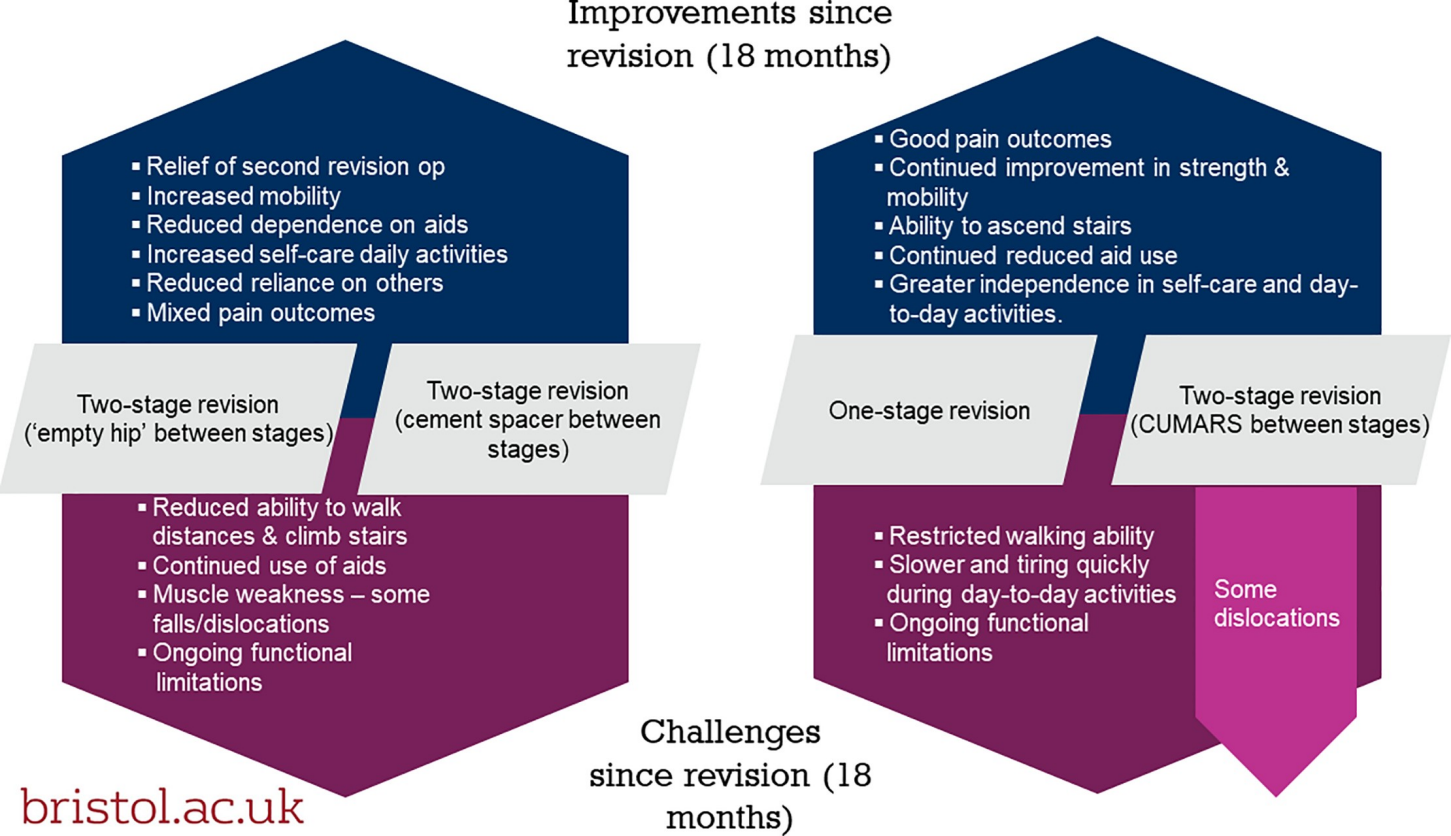
Thematic map of improvements / challenges in patient recovery at 18 months.

Participants described the practical challenges and gains they experienced during day-to-day life while recovering from revision at home. Our analysis identified that the experiences of participants undergoing two-stage revision with an excised hip or a cement spacer differed from those undergoing one-stage revision or two-stage revision with a CUMARS, specifically in relation to mobility, independence and the meaning of the ‘interim’ between revision stages. We also identified experiences of pain and perceived slow recovery that were specific to participants with a cement spacer, or CUMARS respectively (Fig 1). At 18 months post-revision all participants described improvements in mobility and independence, but also ongoing restrictions to walking and some functional limitations (Fig 2).

Regardless of revision type, participants identified needs for information and support during recovery. They also described their emotional resilience and the benefits of their participation in the surgical trial, which contrasts with our previous work with patients who had experienced PJI outside the context of a trial [7]. Themes and subthemes characterising common and contrasting experiences of participants during early and longer-term recovery from revision are described below. Illustrative anonymised data extracts are provided.

### Early recovery experiences

#### Prolonged hospital stay after revision

After one- or two-stage revision most participants remained in hospital for 2 to 4 weeks although some stayed longer due to wound leakage or infection markers remaining high. During this time being informed, and having questions answered by medical or surgical staff were highly valued by patients. However, the prolonged time in hospital was described by some as emotionally wearing.

#### Antibiotic burden

Some participants reported that their long hospital stay was because of the intravenous (IV) antibiotic regime after revision. Several found the regime to be burdensome, requiring them to be immobile for hours at a time during IV administration, which also disrupted sleep and ability to eat. Others were unhappy or unwilling to remain in hospital for the length of stay required while IV antibiotics were administered, several of whom were discharged from hospital to have their IV antibiotics in the community (see below). Most participants experienced unpleasant gastrointestinal side effects from both intravenous and oral antibiotics but thought that such effects were an unfortunate necessity to being infection free.

Eight participants reported that they were discharged from hospital with a Peripherally Inserted Central Catheter (PICC line) and had IV antibiotics in the community. For most this was managed at home by nursing or community teams, although two travelled daily to a treatment centre or hospital. Participants who were discharged in this way were positive about their return home, reduced travel to outpatient appointments, and the opportunities to discuss care and ask for assistance from the health professionals coming into the home.

#### Physiotherapy provision

There was no difference between treatment groups in the provision of physiotherapy. Most participants reported having physiotherapy during their hospital stay but described it as brief and non-personalised; focussed on a small number of activities such as getting out of bed, walking with an aid, and going up steps, after which they were ‘signed off’. Several commented that this predetermined format had not helped them with specific difficulties or functional gains they wanted to achieve. Some undergoing two-stage revision with an excised hip or a cement spacer perceived that physiotherapists had limited understanding of the distinction between primary hip replacement and revision for infection, which they felt led to inappropriate advice for the revision stage they were at. For example, a participant with a non-weight bearing cement spacer was told to put weight on the leg, another reported extreme pain and inability to walk following an outpatient physiotherapy appointment.

About half of participants reported having physiotherapy after discharge from hospital. Access varied with some participants attending a fixed number of sessions in hospital, while others were seen at home by a community physiotherapist. Several reported difficulties accessing physiotherapy, ‘pushing for it’ through their GP surgery after leaving hospital. Participants expressed disappointment that the impetus had been on them to seek out this provision. Varied provision of an exercise plan was reported; in the absence of written materials some participants had repeated exercises given to them following their primary hip operation rather than do nothing. See Table 3 for illustrative extracts.

**Table 3:** Early recovery experiences: Illustrative extracts.

*Antibiotic burden*
George, cement spacer: That's why they kept me in [Treatment Centre 1] for three and a half weeks because I was having intravenous. Err because err we're in the country they didn't think err a err district nurse would be able to come out and give me my intravenous.
Daphne, CUMARS: I was on the intravenous antibiotics for ten days. So I couldn't go home, you know, sort of then.
Steve, one stage: the thing that was keeping me there was that I was on intravenous antibiotics quite a few hours of each day.
Stanley, CUMARS: When I was in [Treatment Centre 3] I was on an antibiotic which started at midnight for two hours and then they switch to another one for a half hour so that was two and a half hours from midnight onwards that I was there with a machine going beep, beep, beep alongside of me, er, lines into my arm which again doesn’t help you sleep.
Albert, one stage: And the other awkward thing was that they always seemed to do [IV antibiotics] when the meal-time comes in… And then you can’t move your hand because you’ve got the IV in and if you moved it because it’s erm on the machine—the machine would stop.
Frank, one stage: I was. . . put on a drip, and which meant for 23 ½ hours, I was just laying there. So, in the end, I, I had to turn round and say, ‘I’m going to discharge myself because there is no, there is no point’. So the consultant came to see me and we agreed that I would go back every day… just for the drip which was a better arrangement, until… home nursing [had] a vacancy to come in and do it.
Elspeth, cement spacer: one of the doctors said I got to stay in there for four weeks while I was having the antibiotics given to me. And that horrified me but fortunately um, in [hometown] they’ve got a team that comes round and gives you the antibiotics at your home. So they set all that up for me, so that was, that was better and that cheered me up and made me feel a lot better, that I could come home.
Ruth CUMARS: they expected me to stay in the hospital another 12 days [for IV antibiotics]. I said, 'I'm not stopping here 12 days.' I said… ‘I had at-home nurses last May… and these nurses came to my home for a fortnight’… And he, he went away to find out and he come back and he said, 'Oh yeah.'
Colin, one stage: when I first started taking them, one of them did give me diarrhoea…. it did sort of settle down, but, erm, yeah.
Jayne, one stage: One of the side effects of amoxicillin is I had a really bad case of thrush.
Winnie, CUMARS: changing from having intravenous antibiotics to having oral ones, which was sort of a view to me being able to go home, I was so violently sick. Not just a little bit sick
Enid, CUMARS: Erm I had a, a PICC line in. [yeah] Erm I wanted to come home… I think I stayed in about a fortnight. But then I came home for the last three… weeks… and the, the nurse came in every day to do it.
*Physiotherapy provision* (In hospital)
Arabella, one stage: If I had any complaint at all […] it would be about the physiotherapy. […] it seems to me whether you’ve had, a revision to your hip or whether you’ve had … just a first, erm, hip replacement, you get exactly the same physiotherapy, and erm, the main aim is, is to get you, know that you can get out of bed safely, which is quite a good thing, and to, to make sure that you can walk and then do the stairs.
Lois, excised hip: I regularly saw physios who, erm, could understand, you know, there wasn’t a lot that could be done, but I had to make sure that the muscles were, were still being worked gradually.
Daphne, CUMARS: They came in very briefly in the ward… I mean it's a matter of about two minutes and that was it. They didn't come back and check that I was doing the exercises.
Steve, one stage: The physio—they were brilliant they you know got me up and going, er, Saturday, er, in the morning walked the length of the ward with the physios [okay], er, er, Sunday […] visit and walk by the physios […] so Monday […], er, physios took me to practice stairs and basically then they signed me off on Monday
Jenny, CUMARS: well I did get up and walk you know once there was, I was discharged very quickly from physio because they got me up, I walked a bit and then they got me up and I could walk a bit further and then I did the stairs, er, and then they discharged me.
Janet, cement spacer: I had quite a bit of physio. I went up those wooden steps and back down again before they'd let me home. [Did they give you any exercises for the home?] Yeah, I had to keep moving my feet and erm bending my legs and—she was very good.
*Physiotherapy provision* (At home)
Arabella, one stage: When I went to the doctor, er, she referred me for emergency physiotherapy in the community, but in actual fact I think probably that should’ve been done from the hospital, so that I could have had it sort of as an on-going thing.
George, cement spacer: a physio has visited me three times [and how's that treatment?] … Very good, yes, they go through the exercises which I've been doing.
Colin, one stage: [Have you seen any physiotherapists at all?] Well only in the hospital of course.
Teresa, CUMARS: When I came out, they didn't give me any exercises to do, but… community physiotherapist came over yesterday so she set me on what I should be doing… I thought, ‘Well, this is what I did last time so it’s what I’ll do this time’.
Sandra, CUMARS: I’d love to see a physio and talk to them and say, is there anything I should be doing to strengthen my muscles or will it overdo this? … you’re not given any support like that, once you leave hospital that’s it, finito.
Jenny, CUMARS: There didn’t seem to be any back up from [Treatment Centre] about going to see a physio.
Trevor, cement spacer: I went to, erm, [city hospital] for some physio and a girl pulled my leg out to the side and, ever since then, I couldn’t walk on it, because every time the foot came flat on the floor, I got a shooting pain up my body.
Fred, cement spacer: Like, when the first physio that come out said, ‘Well you can stand on that.’ I said, ‘No, I can’t.’ She said, ‘Yeah, you can.’ I went, ‘Well, my consultant told me I can’t stand on it… they seem to come in and think everybody’s got a new hip in. They’ve got to get you out doing this and that, and the other, but it doesn’t work that way, I’m afraid.

### Experiences of recovery once home after surgery

#### Participants with an excised hip or cement spacer: Immobility and confinement

When interviewed at the first time point, participants undergoing two stage revision with an excised hip or a cement spacer were positive about ‘feeling better’ than pre-revision, but their accounts were dominated by their severe mobility restrictions which affected virtually every aspect of their daily life. Participants were heavily dependent on mobility aids and described ‘hopping around’ using a walking frame which felt unsteady, leading to anxiety about overbalancing. Participants described the physical effort, time and forethought required to move even small distances or to complete small actions, and the exhaustion this caused.

Due to their immobility, participants described how they were no longer able to undertake activities of daily living previously taken for granted such as self-care or making a cup of tea. Although participants described good provision of equipment to assist them within the home, they described a lack of provision of equipment to help them mobilise outside the home and some were physically unable to get out of their house due to steps or other physical barriers until modifications were made, often by a family member. Despite immobility, four participants reported being told to either buy or rent their own wheelchair because they did not meet the criteria for NHS provision. Participants described frustration and isolation whilst inactive, immobile and confined to the house, and for some this had a heavy emotional toll. The mobility challenges for those with an excised hip or cement spacer also made attendance for blood test or follow-up appointments difficult.

#### Impact on personal and social relationships

As a result of their immobility, participants with an excised hip or a cement spacer described being highly reliant on others to manage many day-to-day activities. For most participants, their partner became responsible for this caring work although some had the help of wider family, friends, or formal care provision. Several reported feeling guilty about the increased burden of caring and household responsibilities on their partner. Many were frustrated by seeing household tasks done to a different standard as their usual roles were disrupted during the treatment and recovery period. Wider family relationships were also impacted as participants’ immobility and confinement led to reduced contact, particularly with grandchildren, and help previously provided for other family members was disrupted by the participants’ heightened needs. See Table 4 for illustrative extracts.

**Table 4:** Participants with an excised hip or cement spacer: Illustrative extracts.

*Immobility and confinement*
Fred, cement spacer: Usually, by the time I get to bed, I’m knackered from going round, and what I do on my Zimmer frame. It’s quite exhausting really.
Arthur, excised hip: I’ve got to just sit in the wheelchair or, or hop about round home like on this frame like.
George, cement spacer: I have to use a Zimmer and I sort of I hop around … I can't go downstairs and I can't go upstairs. When, when I say stairs we've got two steps.
Anna, excised hip: I’m really tired at the end of the day, er, physically and–and emotionally drained a lot of the time, you know, cause I’m having to think continually to minimise, ‘Right, what do I need?’
Cathy, cement spacer: Getting about isn’t easy and being able to do anything. Erm, I really am restricted ‘cause I’m on crutches. And the restriction’s just a bit frustrating from time to time but that’s because I’ve always been so active I think.
Bernie, cement spacer: It’s like taking three months out of your life hasn’t it, you’re just stuck doing nothing.
Janet, cement spacer: Well, I do get very moody. I get frustrated because I can't do things that I want to do. And my husband does all the housework and things like that.
Lois, excised hip: It’s been very difficult because I’m really restricted on what I can do. Erm, because I can only hop round on a gutter frame.
Bernie, cement spacer: I can get [to the kitchen] but I can’t carry it … trying to carry a cup of tea going on this leg, it’s not easy. I can make the tea, I can wipe up, but I can’t put the things away.
Trevor, cement spacer: I can go out and make myself a cup of tea … I can carry it in, but I’m very, very slow bringing it in. I can’t do a bowl of cereal or anything like that. I can’t hold the bowl with the crutch.
Jack, excised hip: I very rarely leave the flat now, you know, because obviously, I can’t walk very far and I’ve got no transport, unless I rely on other people.
George, cement spacer: At the present moment I'm sort of housebound. They are going to do something shortly, I hope, for next week they're going to try and fit me with a wheelchair and a ramp so that I can get outside.
Anna, excised hip: I’m without a hip joint, I’m on two crutches, erm, I’ve been really low at times, extremely low, but I’ve picked myself up now.
Lois, excised hip: for at least two weeks I couldn’t even get out into my back garden and sit in the fresh air because I had no ramp to get me over the French doors into the garden … that was a difficult time, yeah.
*Impact on personal and social relationships*
Fred, cement spacer: I’m relying on my wife, obviously, a lot more than I normally do. [How do you feel about that?] Erm, pretty guilty sometimes.
Bernie, cement spacer: I can’t help her. Normally we share the housework together, I can’t do that.
Janet, cement spacer: Erm I don't like to see my husband doing all the housework, erm because I don't think it's fair on him.
Elspeth, cement spacer: My husband’s had to… finish doing [a part-time job] because I’m having to depend on him all the time. I mean I can’t even get in the shower on my own… I think he’s tired.
Anna, excised hip: [Partner] has to do all [the lawns] and he’s barely got time to do it… and I can’t stand anything that’s in a mess.
Fred, cement spacer: I’m not getting to see [family] so much… at the moment it’d just be too much work.
Bernie, cement spacer: We don’t know [younger grandchild] as much as we know the older one. Which is very sad really because… we haven’t been down there.
Janet, cement spacer: [I’d like] to be able to go out or go and visit my kids, which I haven't been able to do.

#### Participants with one-stage revision or CUMARS: Increased mobility and independence

Most participants undergoing one-stage revision, or two-stage revision with a CUMARS were positive about ‘feeling better’ and in relation to the progress they felt they were making in terms of their mobility and movement. In contrast to those with an excised hip or cement spacer, most described being mobile with aids quickly after the revision operation, and progressing from a walking frame to crutches, or a stick. The majority were mobile within the home and able to leave their home and go out for walks. Most participants also reported being able to undertake some self-care and household activities including washing and dressing themselves, preparing or cooking food and undertaking house or garden work.

#### Ongoing frustrations and reliance on others

However, most also reported some ongoing restrictions and physical limitations. Bending was restricted, which made putting on shoes and socks, dressing their lower half, and foot care difficult. It also limited the ability to pick things up from a low position and to garden or clean as they would like. Although some had returned to driving, nearly all described difficulties getting in and out of cars which made getting to appointments and elsewhere difficult. Although most participants undergoing one-stage or two stage revision with CUMARS appeared to gain independence quickly, there was also variation in ability amongst participants within both treatment groups. A small number reported mobility and functional challenges, including shaky or unsteady walking or feeling insecure because of their CUMARS; as well as difficulties with lifting, carrying and prolonged standing. These few remained heavily dependent on others for household tasks or with aspects of self-care and in common with those with an excised hip or cement spacer, they were concerned about the increased burden of care on their partner.

Several participants with a CUMARS also reported that recovery from their revision operation was slower than recovery following their primary hip replacement. While some put this down to the revision being a bigger operation, others were concerned by what they perceived as poor progress in comparison to their earlier recovery experience. See Table 5 for illustrative extracts.

**Table 5:** Participants with one-stage revision or CUMARS: Illustrative extracts.

*Increased mobility and independence*.
Colin, one stage: I had a Zimmer up until only really last week, I’ve only just got onto–to–I find one stick is easier than two.
Jayne, one stage: I go shopping now with my husband, and we go out for walks, nothing too strenuous. [And how often do you get out and about?] Oh practically every day I try to move about.
Thomas, one stage: I’m walking okay now, erm, I still use the crutches–or one crutch really now. But I find in the house itself, since I was discharged, I’ve come on a lot better than I thought actually.
Freda, one stage: I’m not walking with a frame anymore; I’m walking with two sticks, with one stick in the house. I’ve been up the garden a couple of times.
Winnie, CUMARS: I’m still not really doing very much. I can get around the house quite happily and gardening–as I say, I can stand and prune a tree against, you know, a shrub against a wall.
Alex, one stage: I walked around without using that stick for about three to four hours and I felt pretty positive after that. I thought I could get rid of this crutch because I don’t need it.
Sandra, CUMARS: I’ve got two handrails up the stairs now [right], my right hip is not brilliant and when I go for a walk with the dog I take my two walking poles always… I’m mobile yeah [and] I can do stuff, I can bend, I can stretch, I can get dressed you know everything like that’s fine.
Geoff, one stage: I've been out this morning for a walk, I've been sitting outside in what little bit of sun we had… I'm not but I feel as though I could do without my crutches.
Ian, CUMARS: I can walk on one crutch and I can make myself a cup of tea and a sandwich and I could be, I could be independent if necessary, so I’m not finding anything really challenging around the house.
*Ongoing frustrations and reliance on others*
Alex, one stage: Well the frustration of not being able to do what I could do before really… I mean there are things that I would like to be able to do here in the house. I mean I can do basic stuff like cleaning with the vacuum cleaner round. I can also push a lawn mower about.
Ian, CUMARS: The frustration and killing of time, because I’m the sort of bloke that is generally on the go all the time… I do like my golf and cycling to work and things like that, the thing that you take for granted.
Jenny, CUMARS: Just losing my independence really, not being able to do what I want to do, not being able to go where I want to go, having to rely on other people.
Freda, one stage: I can’t bend over very much. So if I drop anything on the floor; I’ve got a grabber to pick it up and also I’ve got to shout for help.
Colin, one stage: I can’t do the things I used to do at the moment, and–or go where I used to go, erm, but apart from that, erm, well my wife sort of has to do everything at home.
Ruth, CUMARS: I've only just, like I said, started to dress the last three days but erm any chore is hard work. I can't lift anything.
Enid, CUMARS: I've only been out of the house once… I don't feel secure with it… it's perhaps my imagination but it doesn't feel as though it's safe, somehow.
Teresa, CUMARS: I’ve got the reablement, erm, people are in at the moment four times a day. I needed help because I couldn't actually carry the bowls. I couldn't lift the bowl out of the oven.
Daphne, CUMARS: I was getting a little bit concerned that I wasn't making the progress that I thought I might have done, to be honest.
Sandra, CUMARS: I can’t do as much, er, six weeks after I had my [primary] hip you know my walking was, it got better and better every day.
Teresa, CUMARS: I’m finding it alright, but I–I am definitely nowhere near as far forward as I was with the first revision [okay]. Erm, but then I know it was much more–a much more difficult operation

#### Experiences of pain across treatment groups

Most participants with a cement spacer reported that the spacer caused them pain both generally and when in a moving car which jarred the hip. This differed from the experience of participants with an excised hip most of whom described an absence of pain. Most participants undergoing one-stage or two-stage revision with CUMARS also described minimal or no pain. Two participants with a CUMARS reported pain when walking but nonetheless described it as better than before revision. See Table 6 for illustrative extracts.

**Table 6:** Experiences of pain across treatment groups: Illustrative extracts.

George, cement spacer: Err I'm on painkillers and I take them every four hours. So I cope with it.
Cathy, cement spacer: The pain is on movement. Erm, getting up and sitting down. Erm, in the morning it’s particularly painful because I’ve been a long time I suppose without painkillers.
Janet, cement spacer: Erm I've still got quite a bit of pain [from the spacer].
Elspeth, cement spacer: I mean it’s very painful but I am walking about the house and I do go into the garden and, but I can’t walk far.
Trevor, cement spacer: Since I’ve had this, erm, cement [spacer] one put in … I can’t really travel that much. You do literally feel that every, single bump in the road
Jack, excised hip: Once they’d removed the hip, obviously, there was no pain
Arthur, excised hip: [Have you had any pain at all?] Erm, hardly anything really. I haven’t–well, better than I thought really.
Colin, one-stage: I must–I must be honest, I haven’t got a lot of pain.
Thomas, one-stage: I’ve had no pain–none at all really. Not what I call pain, anyway.
Freda, one stage: Everybody says I look a lot better. And, as I say, I am not in pain.
Steve, one stage: The hip now I would say varies, there is sensation, I would not call it pain.
Darren, CUMARS: No pain whatsoever, to be quite honest. The hip has been good as gold.
Teresa, CUMARS: It’s nowhere near as painful as it was before I had the operation.
Ian, CUMARS: Just being pain free is such a wonderful thing.

#### Two-stage revision: Challenging or uncertain times between stages

Participants undergoing two-stage revision with an excised hip or a cement spacer experienced a challenging ‘interim’ between the first and second stage of their revision operation due to the intense restrictions on mobility which participants described as a detachment from life or period of waiting to ‘get back’ to living.

In comparison, participants undergoing two-stage revision with CUMARS reported experiencing uncertainty about whether they would have a second revision operation. Some reported that their surgeon had described patients who had kept their CUMARS for a number of years, which suggested that their CUMARS might last a relatively long time. These participants therefore experienced ongoing uncertainty during the interim period which contrasts with those with an excised hip or cement spacer for whom a second operation was definite. Most participants with a CUMARS said that they would prefer not to have the second operation, with some describing the prospect in negative terms, while many participants with an excised hip or a cement spacer experienced a positive anticipation of the second operation being complete as it marked an end to their detachment from life. See Table 7 for illustrative extracts.

**Table 7:** Two-stage revision: Challenging or uncertain times between stages–Illustrative extracts.

Bernie, cement spacer: l just be glad when it’s over… I can start back living again then and get our life back together but between now and then there’s virtually nothing I can do.
Janet, cement spacer: I want to get back to another life. . . I had a whole year of not being able to do things or do anything that I want—erm I want to get back and have a life.
Anna, excised hip: Hanging on and hanging on, you put your life on hold. . . the impact is immense, immense.
Teresa, CUMARS: [Consultant] also told me that the, erm–some people don't actually have the second stage, they’ve got on so well with the first stage as it is.
Stanley, CUMARS: if it’s a choice… [consultant] said we’ve had people who’ve had these for four or five years, yes that would be nice to not have to have another operation.
Daphne, CUMARS: To be honest, my heart sank when I was told I had to have another operation. I can't bear it, you know.
Winnie, CUMARS: Well, it hangs over you that, you know, this is still looming ahead and then it just sort of sets you back again for another couple of months.

### Perceptions of longer-term recovery: Improvements and losses

At 18 months, five participants who had lived with an excised hip or cement spacer had undergone their second stage revision (See Table 2). They described a reduced dependence on aids, being able to get out of the house, and to do more self-care and household activities; consequently, reliance on others had lessened. However, nearly all also reported the inability to walk any distance, most continued to use a mobility aid and described difficulties lifting, bending and getting in and out of cars. The experience of pain after second stage revision was mixed. Two participants directly attributed muscle weakness in the revised hip to the lengthy interim period (which ranged from 4–7 months) and described falls or dislocations of the new hip which they attributed to this and which complicated their recovery.

Twelve participants who had undergone one-stage, or two-stage revision with a CUMARS described continued improvements in mobility and muscle strength, and a number reported no longer using aids. Many reported ‘doing more’ for themselves in the house and garden. Nearly all reported minimal pain. However, most also described that they were unable to walk distances ‘like they used to’, being slower or becoming fatigued doing day-to-day activities. Difficulties bending and getting in and out of cars remained. Due to their reduced abilities, nearly all participants described having to give up social activities.

Participants’ perspective on the success of their revision and recovery was based on the extent to which they perceived they had ‘returned to normal’ i.e. to pre-infection levels of mobility and function. Consequently, regardless of whether they had undergone one-stage, or two-stage revision with excised hip, cement spacer or CUMARS some participants were positive about their recovery, whereas others were disappointed or concerned that their revision had not returned them to pre-infection levels of ability. See Table 8 for illustrative quotes.

**Table 8:** Perceptions of longer-term recovery—Improvements and losses: Illustrative extracts.

Elspeth, revision after cement spacer: I’m slow, very slow, walking.
Cathy, revision after cement spacer: I’m restricted with distant walking, you know?
Anna, revision after excised hip: The big issue is because I had no hip joint in … for five months the muscles just stopped working properly because I wasn't using them. [yeah] They're still not right.
Elspeth, revision after cement spacer: [I had three dislocations] because I’d had the hip out so long that’s why it was so easy to dislocate it [because the muscles weren’t developed…]
Stanley, kept CUMARS: I once belonged to a walking group. Finished.
Geoff, one-stage: We certainly haven't [gone on holiday] … because I can’t walk very far.
Jenny, kept CUMARS: I was hoping that I would be back to the same as I was after I’d had the first hip done and I’m not, but I thought I would be.

### Emotional resilience during recovery and the therapeutic value of talk

A minority of participants across treatment groups described points during their recovery at which they felt ‘depressed’ or ‘low’, and several described that this had occurred in the early weeks at home after surgery. However, most also described these feelings as short-lived and that being told of encouraging test results such as a reduced CRP level, or physical signs of recovery or making functional progress had a positive emotional impact on them. One participant who had suffered frequent reinfections and was living with ongoing pain described themselves as being in a depressed state.

Although it was common for participants in all treatment groups to describe frustration in relation to mobility and activity restrictions, the majority emphasised that they had not been negatively emotionally impacted during their recovery from PJI. Many credited their social support network or their own resilient attitude for their positive outlook. Participants also emphasised the importance of interactions with surgical and medical staff in which questions were answered and information shared and explained to them. A number contrasted this with earlier hospital stays where they felt they were not as well informed. It appeared that participation in the research trial had increased the opportunities for these positive interactions for many participants. Several described that they enjoyed and highly valued the opportunities to talk to the research team during the trial; one felt that these opportunities to talk had been beneficial to her recovery, and another had found being able to explain her experience face-to-face more rewarding than completing the formal questionnaire study tools. Others valued the ongoing contact and monitoring from the trial study team and found this to be supportive and reassuring during their recovery. See Table 9 for illustrative extracts.

**Table 9:** Emotional resilience during recovery and the therapeutic value of talk: Illustrative extracts.

Jayne, one stage: I mean I have gone through patches of feeling quite low … the house has been sort of moved around to accommodate me, and it looked untidy, there was things I couldn’t do you know like cleaning … which is frustrating.
Alex, one stage: I was [down] because of the CRP. I am a little bit still because of the erm–the lack of the mobility and the frustration I get. But I’m more upbeat now, particularly with the infection/inflammation level [reducing].
Anna, excised hip: once I'd worked things out and realised I could do things a little bit more … then I gradually started to feel a bit better [yeah, yeah] and got a bit more used to it.
Elspeth, cement spacer: I couldn’t even get in the car to be taken out for a ride, being in the house all the time … I understood the situation but it did make me feel very down, tearful and that’s not me, no.
Darren, CUMARS: [do you have any feelings of feeling down?] No, none at all really, apart from, well, it’s not down; I just feel, well, frustrated.
Trevor, cement spacer: I wouldn’t say I’ve been feeling low because when I’m awake I’ve been doing crosswords and things like that.
Steve, one stage: But from the hip no I’ve got no depression at all, never have had.
Enid, CUMARS: No, it doesn’t affect me. I’m too old to have them airy-fairy ideas. What is what is and I can’t do anything about it.
George, cement spacer: I'm not depressed or anything at all, no, I never have been… When I went to [local hospital] first time and they couldn’t control [the infection] … then I got a bit depressed because nobody was telling me. But in [Treatment Centre 1] … if I wanted to know anything I asked and I got an answer.
Christopher, one stage: The [consultant] … was really good at explaining the whole procedure and what had happened … put my mind at rest.
Janet, cement spacer: In the [Treatment Centre] they were very good. They explained everything to me.
Arabella, one stage: Just being able to talk things through rather than just I think it probably leaves you as a healthier person than if you don’t take part in the [research].
Anna, excised hip: I’d rather talk to somebody … over the telephone, face to face is nice as well […] because that–that contact, erm, you can express more than whatever you can on a piece of paper and replying to set questions … I feel they didn’t exactly apply to me in that questionnaire that was sent out.
Jenny, CUMARS: I was really pleased … that we’ve had, you know, these talks and I’ve had to have these forms to fill in and things. I was pleased about that because I felt as though I wasn’t being just left.
Elspeth, cement spacer: I mean I wanted to know how, how life was going to be um, after I’d had, when I hadn’t got the hip. And [research nurse] explained it all to me and what she’s said is right.
Lois, excised hip: I do enjoy the fact that, erm, people keep checking on me [okay], I think that’s very helpful.
Sandra, CUMARS: It’s been nice talking to the people like [researcher].

### Support and information needs during recovery

Participants in all treatment groups wanted information at key points both before and throughout their treatment and recovery. At the time of their primary hip replacement participants wanted to be made aware of infection as a complication of hip replacement and informed of the symptoms of infection. When being treated for infection participants undergoing two-stage revision with an excised hip or a cement spacer wanted information about what to expect during the period between stages, including practical information about mobility and travel challenges, such as how to source a wheelchair and access hospital transport for appointments. Participants across treatment groups wanted information about how recovery from revision surgery was likely to differ from recovery after primary hip replacement in order to guide expectations of progress. Guidance about how to avoid dislocation was also desired.

Throughout their recovery, all participants wanted physiotherapy intervention or advice tailored to their specific physical needs, perceiving that this would support their physical recovery and offer reassurance about progress. Participants also articulated a general need for contact with a fellow patient or health professional who was knowledgeable about PJI and revision during their recovery in order to share experiences and offer mutual practical and emotional support. The early weeks at home after revision were identified as an important time at which such contact would be valued, possibly because of the sudden and abrupt change in mobility and loss of independence.

## Discussion

This study identifies both commonalities and differences in the recovery experiences of participants undergoing one-stage and two-stage treatment for PJI and finds that recovery experience may differ between those undergoing two-stage revision with an excised hip, cement spacer, or CUMARS. The main difference identified was that participants undergoing two-stage revision with excised hip or a cement spacer during the interim period faced extreme restrictions in their day-to-day activities and confinement to the home which resulted in frustration, high reliance on others, and potential isolation. This resonates with the earlier finding that patients undergoing both one- and two-stage revision experience mobility and lifestyle limitations, and that the intensity of these are heightened for patients undergoing an interim between operations [17]. Due to their mobility and functional restrictions, we found that participants with excised hip or cement spacers experienced the interim as a challenging period of waiting during which they felt detached from life. Dependence on others impacted and potentially changed relationships as roles were disrupted and maintenance of wider family bonds became more difficult.

By comparison, participants undergoing one-stage, or two-stage revision with a CUMARS broadly experienced greater mobility, physical range and ability with day-to-day activities during recovery, and less pain, although participants did vary within these groups. However, these participants also experienced frustrating restrictions to their function which limited their activities and led to varying degrees of reliance on others.

These findings echo the impact on family and relationships, and the ‘changed life’ following infection and treatment for PJI identified previously [7]. However, we also found emotional resilience amongst participants undergoing either one or two-stage revision which contrasts with the psychological difficulties faced by patients undergoing revision surgery identified by previous studies [7, 13, 18]. We suggest this is linked to participants’ involvement in the trial, as they reported that the contact and opportunity to talk with members of the research team had a supportive and beneficial emotional impact during recovery from revision. This links with the finding that monitoring patients’ recovery is in itself an important component in rehabilitation after joint replacement [19].

Our study is the first to draw attention to the differences in the lived experiences of those with a with or without spacers of different kinds and how these affect patients’ recovery experience. Our findings add to the evidence base by characterising the common experience of pain amongst participants with a cement spacer, which appeared to differ from the experience of patients undergoing other revision treatments. This extends previous research which shows frequent mechanical complications with cement spacers [20]. We also identify that participants undergoing two-stage revision with a CUMARS experience ongoing uncertainty during the interim and may experience less positivity about the prospect of a second operation or perceive that progress in mobility and functional recovery is slow by comparison to primary hip replacement.

All participants identified a need for emotional support and specialised physiotherapy intervention during recovery, as well as better information provision about what to expect during the interim after revision which is in keeping with previous research [7, 17]. However, our findings lend weight to this need as those who took part in the trial identified that increased opportunities to talk, receive information and share experiences had an emotionally beneficial effect during recovery. We also identified that discharge with a PICC line may reduce the burden of a prolonged hospital stay on participants. At 18 months post-revision, participants’ perception of their ‘recovery’ varied and most reported that their ability to walk, and some functional abilities remained diminished, which confirms the previous finding that overall levels of improvement are lower after revision surgery than after primary hip replacement [21]. Some participants were disappointed not to have returned to their pre-infection abilities. The lasting impact of PJI and revision was therefore life changing [22].

Interview data were generated with a large sample of 32 participants from across 8 clinical sites, ensuring that a diverse range of experiences and perspectives were included, thereby enhancing the potential for transferability of the results. While diversity in a sample may be seen as a limitation within other research approaches that seek to minimize variables when comparing interventions, it is a strength of qualitative research that seeks to understand a range of experiences and perspectives. Additionally, the achievement of saturation, such that no new themes were emerging highlights the concordance across the dataset and also increases the likelihood of the transferability of results to other contexts. The interviews undertaken 2–4 months after revision surgery allowed participants to describe their early experiences of recovery with minimal recall bias while second interviews 18 months after revision allowed longer-term experiences of recovery to be explored. Dual coding by two researchers and undertaking comparative analysis between treatment groups ensured rigor. However, our ability to compare experiences of recovery at both post-operative and 18-month timepoints in the same patients was limited due to being unable to re-interview all 30 of the original participants at the 18-month timepoint. We did however, interview other participants at this timepoint to balance the characteristics of the sample and to ensure saturation. We also acknowledge that participants who took part in the qualitative study may have been those who were most positive about trial participation.

Overall, our study suggests that recovery experiences after one- and two-stage revision are characterised by both gains and losses in mobility and function, and that experiences may differ in some respects in relation to the use or non-use of spacer and the spacer type. Clinical measures of success following one- and two-stage revision have traditionally been reinfection and/or mortality rates at a timepoint after completion of surgical treatment [9, 10]. This study identifies that mobility, function, independence and pain are important aspects of the patient experience that affect all areas of their day-to-day life. The care pathway for those experiencing PJI would be improved with greater provision of tailored physiotherapy, and increased opportunities for talk which may be emotionally beneficial whilst also providing practical information.

## Supporting information

S1 File (PDF)

S2 File (PDF)
