# Supplementary material for: Post-surgery and recovery experiences following one- and two-stage revision for prosthetic joint infection—A qualitative study of patients’ experiences
Source: PLoS One. 2020 Aug 3;15(8):e0237047. doi: 10.1371/journal.pone.0237047 (PMC7398523; doi:10.1371/journal.pone.0237047)
Supplement: S1 File — (PDF) [file pone.0237047.s001.pdf]

S1. Patient Topic Guide: Timepoint 1

Introduction and consent: Re-affirm the verbal consent process and check agreement

Discuss how the interview will be recorded, and reassure the participant about issues of confidentiality and anonymisation.

Aim of the study: to understand participants' experiences of taking part in the trial, and their experiences of treatment for infection after joint replacement.

Socio-demographic data

- marital status / employment status / dependents / hobbies / living situation / other health conditions / needs / previous surgery?

Understanding and acceptance of the recruitment process

- Can you tell me when you first heard about the INFORM project? How did you hear about the trial?
- How did you feel about taking part in the trial?
- Did you have any concerns about taking part? *Can you tell me more about those?*
- Who explained the trial to you? *How did you feel about the trial at that point?*
- Did your surgeon explain the trial to you? *How did you feel about the trial at that point?*
- What did you think of the written information that you received about the trial? *The information booklet?*
- Can you remember if you asked any questions about the project?
- Did someone come to talk to you about the trial? *Did you feel you understood the information given to you (language used, terminology*)?
- What do you understand about the term 'randomisation'?
- How did you feel about being randomised to one treatment or the other?
- How have you found your participation in the trial so far?
- Have you found anything particularly difficult, or inconvenient?
- Is there any part of the trial that you have enjoyed so far or feel positive about?
- How do you feel about being treated at the Avon Orthopaedic Centre / Cardiff / Exeter / Sheffield/Oxford/Oswestry?

Experience of treatment

- Can you tell me what kind of surgery you have had for your treatment? *One or two stage?*
- Did you have any concerns about having the revision surgery? *Can you tell me about those*?
- What did you think the treatment would involve? *Did your expectations match this?*
- How were you after the operation?

- Can you remember how long you were in hospital for after the operation?
- Can you tell me about your time in hospital after the operation? What happened?
- Did you have any visitors? Partner, family, friends to visit you while you were in hospital? *Did they have to travel far?*
- What impact has the treatment had on your life so far? *Work / leisure / family / finance?*
- How have you managed since being discharged from hospital? *pain / mobility / self-care / anxiety or depression / sleep*
- IF TWO STAGE - have you had a spacer fitted? *How are you finding it / Is it comfortable / what is your mobility like e.g. can you make yourself a cup of tea? Are you aware of the spacer? Are you having to be careful when moving around?*
- IF TWO STAGE - I realise that you are waiting for the second operation and I was wondering how you are finding this interim period?
- What are you finding most challenging at this point?
- Are you able to get out and about?
- Has there been any complications at all? *(DVT clots, pulmonary oedema, dislocation, nerve damage?) Can you tell me about those?*
- How has your hip infection and subsequent treatment affected other family members or people who care for you? *Partner, children?*
- Can you tell me about the antibiotics you've been given? *Have they had any side effects? Are you managing to take them as prescribed?*
- Do you feel there is anything that could be done to help support you or your family/carer(s) more at this point?
- Do you have any concerns about the next few months in regards to any aspect of your hip or the care that you receive?
- Is there any part of the trial that you have enjoyed or felt positive about?

If you agree I would like to follow your progress and recovery and wondered if it would be possible to talk to you again towards the end of the study approximately 16 months from now? This would be a face to face interview. Would it be ok if I contacted you again about this nearer the time?

Conclusion

- Is there anything else you would like to add, or anything you wish to talk about that we haven't covered already?

Reaffirm consent…thank you for participating…END.
