# Supplementary material for: Post-surgery and recovery experiences following one- and two-stage revision for prosthetic joint infection—A qualitative study of patients’ experiences
Source: PLoS One. 2020 Aug 3;15(8):e0237047. doi: 10.1371/journal.pone.0237047 (PMC7398523; doi:10.1371/journal.pone.0237047)
Supplement: S2 File — (PDF) [file pone.0237047.s002.pdf]

S2. Patient Topic Guide: Timepoint 2

Introduction and consent:

Discuss informed consent, how the interview will be recorded, and reassure the participant about issues of confidentiality and anonymisation.

Aim of the study: to understand people's experiences of participating in the INFORM trial and their experiences of treatment for infection after joint replacement and their subsequent recovery and the impact of these events on their lives.

Socio-demographic data

- Date of birth / marital status / employment status / dependents / hobbies / living situation / other health conditions / needs / previous surgery?

RECAP for patients interviewed at time point 1:

1. Summarise how they came to participate in the trial, how they felt about being randomised, and their experiences of treatment.
2. Check summary is a fair representation of their experience.

"So the last time we spoke you had just had your revision operation / were in the between stages of the 2 stage revision, so can we pick up from there…?" – Move on to "Experience of treatment" section.

EXTENDED for patients not interviewed at time point 1 - Experience of participating in the trial

- EXTENDED - Can you tell me when you first heard about the INFORM project?
- EXTENDED - Can you remember how you felt initially about taking part in the trial?
- EXTENDED – Who explained the trial to you? *How did you feel about the trial at that point?*
- EXTENDED - Did your surgeon explain the trial to you? *How did you feel about the trial at that point?*
- EXTENDED - Can you remember if you asked any questions about the project?
- EXTENDED - Did you have any concerns about taking part? *Can you tell me more about those?*
- EXTENDED - Did you feel that you understood all of the information that you were given about the trial?
- EXTENDED - What do you understand about the term 'randomisation'?
- EXTENDED - How did you feel about being randomised to one treatment or the other?

Experience of treatment

- 2 STAGE – What did you find most challenging about the period between the stages?
- 2 STAGE - Can you tell me about the second operation and how that went? *How long were you in hospital afterwards?*

- 2 STAGE – Do you feel that your family have been affected by these events as well? *What have they found most challenging?*
- Did you have any concerns about having the revision surgery? *Can you tell me about those*?
- Can you tell me about your time in hospital after the operation?
- Can you tell me about your time at home once you had been discharged after your [final] operation? *How did it affect you physically / mentally?*
- In terms of your treatment and care, what do you think was done well?
- Do you feel that there are any areas for improvement in regard to how you've been cared for by health professionals?
- How do you feel about being treated at the Avon Orthopaedic Centre / Cardiff / Exeter / Sheffield/Oxford/Oswestry?

Your recovery since having your treatment

- How did you manage once you were discharged from hospital?
- What have you found most challenging during your recovery?
- Has there been any complications at all? *(DVT clots, pulmonary oedema, dislocation, nerve damage?) Can you tell me about those?*
- How has your general health been? *(Is there anything you would attribute to your treatment?)*
- Can you tell me about the antibiotics you've been given? *Have they had any side effects? Are you managing to take them as prescribed?*
- What impact has the treatment had on your life so far? *Work / leisure / family / finance?*
- Do you feel there is anything more that could have been done to help support you during your recovery?
- Do you have any concerns about the future in regards to your hip?

Feedback on trial experience and conclusions

- How do you feel now about having taken part in the project as a whole?
- Is there anything about your participation in this research that you would have liked to have happened differently?
- Have you found anything particularly difficult, or inconvenient?
- Is there any part of the project that you have enjoyed or felt positive about?

- Is there anything else you would like to add, or anything you wish to talk about that we haven't covered already?

Reaffirm consent…thank you for participating…END.

***2 STAGE – for participants who received a two stage operation**

***EXTENDED – for participants who have not taken part in a previous interview at time point 1**
